# Personalized Lifestyle Medicine: Relevance for Nutrition and Lifestyle Recommendations

**DOI:** 10.1155/2013/129841

**Published:** 2013-06-26

**Authors:** Deanna M. Minich, Jeffrey S. Bland

**Affiliations:** Personalized Lifestyle Medicine Institute, 800 Fifth Avenue, Suite 4100, Seattle, WA 98104, USA

## Abstract

Public health recommendations for lifestyle modification, including diet and physical activity, have been widely disseminated for the prevention and treatment of disease. These guidelines are intended for the overall population without significant consideration for the individual with respect to one's genes and environment. Personalized lifestyle medicine is a newly developed term that refers to an approach to medicine in which an individual's health metrics from point-of-care diagnostics are used to develop lifestyle medicine-oriented therapeutic strategies for improving individual health outcomes in managing chronic disease. Examples of the application of personalized lifestyle medicine to patient care include the identification of genetic variants through laboratory tests and/or functional biomarkers for the purpose of designing patient-specific prescriptions for diet, exercise, stress, and environment. Personalized lifestyle medicine can provide solutions to chronic health problems by harnessing innovative and evolving technologies based on recent discoveries in genomics, epigenetics, systems biology, life and behavioral sciences, and diagnostics and clinical medicine. A comprehensive, personalized approach to medicine is required to promote the safety of therapeutics and reduce the cost of chronic disease. Personalized lifestyle medicine may provide a novel means of addressing a patient's health by empowering them with information they need to regain control of their health.

## 1. Introduction

**Figure 1:**
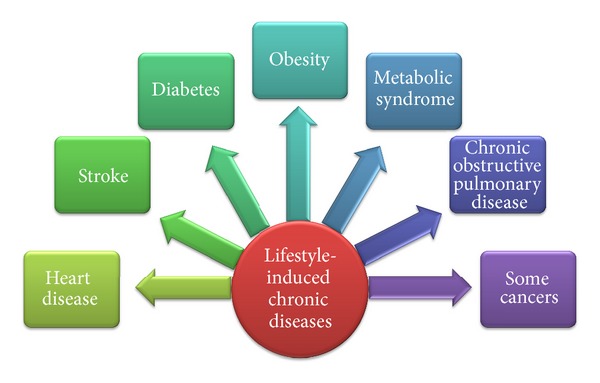
Lifestyle-induced chronic disease.

Chronic conditions such as type 2 diabetes, cardiovascular disease, metabolic syndrome, and obesity are, in varying degrees, associated with unhealthy lifestyle and behavior, including tobacco use, nutritional excesses, lack of physical activity, and heightened exposure to stress (Figure 1). Egger et al. [1] reported that most (about 60%–70%) health care visits in industrialized countries are correlated with these lifestyle-induced, preventable diseases. Therefore, due to the exorbitant cost and lack of resources to deal with the rising tide of illness, the importance of lifestyle factors in the origin and progression of disease can no longer be ignored. In fact, Dysinger [2] documented that in 2012, the American Medical Association issued a call to action for physicians to “acquire and apply the 15 clinical competencies of lifestyle medicine, and offer evidence-based lifestyle medicine interventions as the first and primary mode of preventing and, when appropriate, treating chronic disease within clinical medicine.”

In 2010, Lianov and Johnson [3] published an article in the *Journal of the American Medical Association* that strongly advocated physician education and training in lifestyle medicine: “Physician educators at both the undergraduate and graduate medical education levels should consider incorporating the relevant lifestyle medicine competencies into education and training programs.” The need for education in lifestyle medicine is so profound that prominent universities like Harvard, Stanford, and Yale have implemented the inclusion of lifestyle medicine into their curriculum, ranging from postgraduate courses to the development of separate institutes devoted to the cause. Additionally, the *American Journal of Lifestyle Medicine *is a peer-reviewed journal that was launched in 2007 for the purpose of educating practitioners on how to incorporate lifestyle medicine into clinical practice.

Lifestyle medicine is not a new or alternative medical discipline. The value of food as medicine was acknowledged several centuries ago by Hippocrates. Despite the fact that lifestyle recommendations have been recognized in ancient healing traditions over centuries, there are several modern-day definitions of lifestyle medicine that have been proposed. Dysinger [2] states it succinctly that lifestyle medicine is “the application of simple, natural healing approaches to chronic disease and prevention.” In his textbook on lifestyle medicine, Egger refers to lifestyle medicine as “the application of environmental, behavioural, medical and motivational principles to the management of lifestyle-related health problems in a clinical setting [4].” The Lifestyle Medicine Competency Development Panel defines it as “the evidence-based practice of helping individuals and families adopt and sustain healthy behaviors that affect health and quality of life [2].” Dean Ornish, touted as the most well-recognized pioneer in lifestyle medicine, states that it is composed of nutrition, physical activity, stress reduction and rest, and social support systems [3].

For the purposes of this review, lifestyle medicine is considered in the broadest sense as follows:

- *nutrition,* as it relates to dietary supplements, medical foods, and functional foods;
- *physical activity* as defined by the entire spectrum of movement, from anaerobic to aerobic, and from mild to vigorous in intensity;
- *sress management and behavioral modification,* as needed, and all the aspects that modulate behavior such as mind-body medicine, psychosocial influences, and social networks;
- *environmental exposure* to contaminants found in air, food, water, and radiation, due to the ubiquitous nature of toxins and their compounding concentration in the environment, leading to the well-recognized increasing toxin burden in physiological systems that relates directly to chronic disease.

*Aspects of lifestyle medicine are as follows:*

- whole foods and dietary patterns;
- specific food-derived bioactives;
- liquids and hydration;
- dietary supplements;
- medical foods;
- functional foods;
- physical activities (aerobic or anaerobic);
- mental fitness;
- emotional regulation;
- mind-body medicine modalities for stress modulation;
- social networks and support groups;
- rest and sleep;
- environmental exposures (air, food, water or radiation).

**Figure 2:**
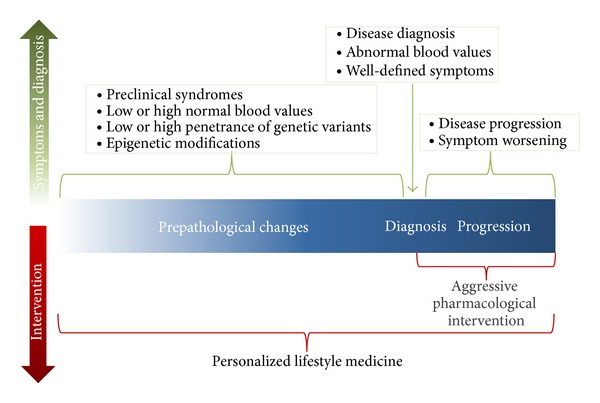
The trajectory of disease and role for personalized lifestyle medicine.

Perhaps most importantly, lifestyle medicine is intended to be patient focused, enabling and requiring patients to be intimately involved in their health trajectory with the accompaniment of healthcare professionals. The system of mainstream medicine is designed for the “typical” patient with lab biomarkers within specific ranges; however, there is a subset of patients who tend to be outliers in this distribution of the population. In fact, these outliers may occur more frequently than is perhaps acknowledged, and, at the same time, they may experience increased difficulty with navigating the healthcare system. Even genetic variants with low penetrance can have significant effects in one's physiology, resulting in low tolerance to certain environmental influences. Furthermore, laboratory values in the low or high normal range may signify the onset of subclinical pathological syndromes, and, ultimately, these individuals may become excellent candidates for what personalized lifestyle medicine has to offer (Figure 2).

In conjunction with being a compelling solution to the chronic disease epidemic and allowing the patient to have control of their health, lifestyle medicine therapies have been shown to be cost effective. Herman et al. [5] assessed both lifestyle intervention and metformin against placebo intervention in the prevention of type 2 diabetes in individuals with impaired glucose intolerance. Lifestyle delayed the onset of type 2 diabetes by 11 years and metformin by 3 years compared with placebo. Additionally, the cost per quality-adjusted life-years (QALYs) was much lower for the lifestyle intervention relative to the implementation of metformin therapy (cost per QALY: $1,100 versus $31,300, resp.). Thus, lifestyle costs less and performs better in one of the largest, growing chronic diseases in developed countries, type 2 diabetes.

## 2. Public Health Recommendations for Nutrition and Lifestyle

Several opinion leader organizations have published lifestyle medicine recommendations consisting primarily of diet and physical activity benchmarks for the general public for the purpose of either prevention or as part of a treatment for diseases. For example, in 2010, the U.S. Department of Agriculture and the U.S. Department of Health and Human Services published extensive guidelines for the American population on what constitutes a healthy dietary pattern, supporting the widespread incorporation of nutrient-dense foods and beverages in proper amounts into the average American diet to assist in maintenance of body weight [6]. For those individuals interested in specific measures for cardiovascular disease risk reduction, the American Heart Association Nutrition Committee published a paper on both diet and lifestyle recommendations [7]. Together with highlighting the benefits of eating a diet rich in vegetables and fruits, as well as eating oily fish twice per week, they discuss lifestyle habits such as smoking cessation and the importance of physical activity in maintaining ideal body weight. According to Kushi et al. [8], the American Cancer Society nutrition and physical activity guidelines are consistent with those defined by the American Heart Association and the 2010 Dietary Guidelines for Americans, in addition to those established by the American Diabetes Association and the 2008 Physical Activity Guidelines for Americans.

*Lifestyle Medicine Recommendations by National Opinion Leader Organizations. *For the U.S. Department of Agriculture and the U.S. Department of Health and Human Services [6], the lifestyle medicine recommendations for general population are as follows.

*Balancing Calories to Manage Weight.*

- Prevent and/or reduce overweight and obesity through improved eating and physical activity behaviors.
- Control total calorie intake to manage body weight. For people who are overweight or obese, this will mean consuming fewer calories from foods and beverages.
- Increase physical activity and reduce time spent in sedentary behaviors.
- Maintain appropriate calorie balance during each stage of life—childhood, adolescence, adulthood, pregnancy and breastfeeding, and older age.

*Foods and Food Components to Reduce.*

- Reduce daily sodium intake to less than 2,300 milligrams (mg) and further reduce intake to 1,500 mg among persons who are 51 and older and those of any age who are African American or have hypertension, diabetes, or chronic kidney disease. The 1,500 mg recommendation applies to about half of the US population, including children, and the majority of adults.
- Consume less than 10 percent of calories from saturated fatty acids by replacing them with monounsaturated and polyunsaturated fatty acids.
- Consume less than 300 mg per day of dietary cholesterol.
- Keep trans fatty acid consumption as low as possible by limiting foods that contain synthetic sources of trans fats, such as partially hydrogenated oils, and by limiting other solid fats.
- Reduce the intake of calories from solid fats and added sugars.
- Limit the consumption of foods that contain refined grains, especially refined grain foods that contain solid fats, added sugars, and sodium.
- If alcohol is consumed, it should be consumed in moderation—up to one drink per day for women and two drinks per day for men—and only by adults of legal drinking age.

*Foods and Nutrients to Increase.* Individuals should meet the following recommendations as part of a healthy eating pattern while staying within their calorie needs.

- Increase vegetable and fruit intake.
- Eat a variety of vegetables, especially dark-green and red and orange vegetables and beans and peas.
- Consume at least half of all grains as whole grains. Increase whole-grain intake by replacing refined grains with whole grains.
- Increase intake of fat-free or low-fat milk and milk products, such as milk, yogurt, cheese, or fortified soy beverages.
- Choose a variety of protein foods, which include seafood, lean meat and poultry, eggs, beans and peas, soy products, and unsalted nuts and seeds.
- Increase the amount and variety of seafood consumed by choosing seafood in place of some meat and poultry.
- Replace protein foods that are higher in solid fats with choices that are lower in solid fats and calories and/or are sources of oils.
- Use oils to replace solid fats where possible.
- Choose foods that provide more potassium, dietary fiber, calcium, and vitamin D, which are nutrients of concern in American diets. These foods include vegetables, fruits, whole grains, and milk and milk products.

For the American Heart Association Recommendations for Cardiovascular Risk Reduction [7], the lifestyle medicine recommendations for general population are as follows.

- Balance calorie intake and physical activity to achieve or maintain a healthy body weight.
- Consume a diet rich in vegetables and fruits.
- Choose whole-grain, high-fiber foods.
- Consume fish, especially oily fish, at least twice a week.
- Limit your intake of saturated fat to 7% of energy, trans fat to 1% of energy, and cholesterol to 300 mg per day by
  - choosing lean meats and vegetable alternatives;
  - selecting fat-free (skim), 1%-fat, and low-fat dairy products;
  - minimizing intake of partially hydrogenated fats.
- Minimize your intake of beverages and foods with added sugars.
- Choose and prepare foods with little or no salt.
- If you consume alcohol, do so in moderation.
- When you eat food that is prepared outside home, follow the AHA Diet and Lifestyle Recommendations.

For the American Cancer Society Guidelines on Nutrition and Physical Activity for Cancer Prevention [8], the lifestyle medicine recommendations for general population are as follows.

- Achieve and maintain a healthy weight throughout life.
- Be as lean as possible throughout life without being underweight.
- Avoid excess weight gain at all ages. For those who are currently overweight or obese, losing even a small amount of weight has health benefits and is a good place to start.
- Engage in regular physical activity and limit consumption of high-calorie foods and beverages as key strategies for maintaining a healthy weight.
- Adopt a physically active lifestyle.
- Adults should engage in at least 150 minutes of moderate intensity or 75 minutes of vigorous intensity activity each week, or an equivalent combination, preferably spread throughout the week.
- Children and adolescents should engage in at least 1 hour of moderate or vigorous intensity activity each day, with vigorous intensity activity occurring at least 3 days each week.
- Limit sedentary behavior such as sitting, lying down, watching television, or other forms of screen-based entertainment.
- Doing some physical activity above usual activities, no matter what one's level of activity, can have many health benefits.
- Consume a healthy diet, with an emphasis on plant foods.
- Choose foods and beverages in amounts that help achieve and maintain a healthy weight.
- Limit consumption of processed meat and red meat.
- Eat at least 2.5 cups of vegetables and fruits each day.
- Choose whole grains instead of refined grain products.
- If you drink alcoholic beverages, limit consumption.
- Drink no more than 1 drink per day for women or 2 per day for men.

For the American Diabetes Association [9], the lifestyle medicine recommendations for general population are as follows.

*Energy Balance, Overweight, and Obesity.*

- In overweight and obese insulin-resistant individuals, modest weight loss has been shown to improve insulin resistance. Thus, weight loss is recommended for all such individuals who have or are at risk for diabetes.
- For weight loss, either low-carbohydrate or low-fat calorie-restricted diets may be effective in the short term (up to 1 year).
- For patients on low-carbohydrate diets, monitor lipid profiles, renal function, and protein intake (in those with nephropathy), and adjust hypoglycemic therapy as needed.
- Physical activity and behavior modification are important components of weight loss programs and are most helpful in maintenance of weight loss.
- Weight loss medications may be considered in the treatment of overweight and obese individuals with type 2 diabetes and can help achieve a 5%–10% weight loss when combined with lifestyle modification.
- Bariatric surgery may be considered for some individuals with type 2 diabetes and BMI 35 kg/m^2^ and can result in marked improvements in glycemia. The long-term benefits and risks of bariatric surgery in individuals with prediabetes or diabetes continue to be studied.

*Preventing Diabetes (Primary Prevention).*

- Among individuals at high risk for developing type 2 diabetes, structured programs that emphasize lifestyle changes that include moderate weight loss (7% body weight) and regular physical activity (150 min/week), with dietary strategies including reduced calories and reduced intake of dietary fat, can reduce the risk for developing diabetes and are therefore recommended.
- Individuals at high risk for type 2 diabetes should be encouraged to achieve the USDA recommendation for dietary fiber (14 g fiber/1,000 kcal) and foods containing whole grains (one-half of grain intake).
- There is not sufficient, consistent information to conclude that low-glycemic load diets reduce the risk for diabetes. Nevertheless, low-glycemic index foods that are rich in fiber and other important nutrients are to be encouraged.
- Observational studies report that moderate alcohol intake may reduce the risk for diabetes, but the data do not support recommending alcohol consumption to individuals at risk of diabetes.
- No nutrition recommendation can be made for preventing type 1 diabetes.
- Although there are insufficient data at present to warrant any specific recommendations for prevention of type 2 diabetes in youth, it is reasonable to apply approaches demonstrated to be effective in adults, as long as nutritional needs for normal growth and development are maintained.

## 3. The Emergence of Science to Support Personalized Nutrition Interventions

Based on this cursory review of national guidelines, it would seem that major health organizations are disseminating essentially similar nutrition and lifestyle recommendations to the public for the purpose of disease prevention. However, it is worthwhile to question whether these standardized public health positioning statements are sufficient to meet the diversity of the average individual, including addressing the multitude of variables such as age, lifecycle, gender, medical history, family history, vitamin and mineral status, ethnic background(s), lifestyle habits, genetics, single nucleotide polymorphisms (SNPs), mutations, and epigenetics. The following are the patient characteristics to consider in establishing a personalized lifestyle medicine therapeutic protocol:

- age,
- lifecycle,
- gender,
- past medical history,
- family history,
- ethnic background and ancestry,
- lifestyle habits (e.g., smoking, activity, and stress reduction practices),
- nutritional status (e.g., macronutrients, micronutrients, phytonutrients, and vitamins),
- medication use,
- dietary supplement use,
- physical location and frequency of travel,
- home and environment,
- genetics and mutations,
- single nucleotide polymorphisms (SNPs),
- epigenetic patterns.

Xie et al. [10] illustrate the complexity of the individual by suggesting the role of metabonomics in personalized nutrition. Metabonomics, or assessment of metabolic responses based on nutrient sufficiency or deficiency, is a way to characterize the metabolic phenotype of individual and predict their corresponding interactions with gut microbiota, environment, and behavior. In addition, Xie et al. [10] discuss advances in the role of phytochemical modulation of cellular physiology and propose phytochemical profiling, or phytoprofiling, to assist in the facilitation of determining phytonutrient requirements with more effective interventions with plant-derived compounds. Hence, the needs of the individual can be complex and require in-depth assessment before interventions can be confidently applied.

In much the same way, determining how the food intake as part of a dietary intervention meets the needs of an individual can be equally daunting. The challenge in understanding the integration of the many facets of the individual with the diversity of food constituents is supported by Jacobs and Tapsell [11] who state that reducing dietary recommendations to individual nutrients without considering the whole food and its multitude of constituents, including phytonutrients, may not be accounting for “food synergy.” Certainly, it would seem that the mere presence and interplay of complex constituents in food would be important to acknowledge in the formulation of dietary recommendations. Improved quantitation of phytochemicals, secondary metabolites, bacterial species, and micronutrients would be useful in positioning of plant foods for their antioxidant, anti-inflammatory, and anticancer properties. Along similar lines, Minich and Bland [12] discussed the importance of phytochemicals from cinnamon, hops, green tea, berberine, ginseng, quercetin, and resveratrol, in the modulation of the intracellular signals related to metabolic processes specific to insulin sensitivity, referred to as selective kinase response modulators, since these phytochemicals amplify signals through the cell selectively via protein kinases and to the degree required to restore intracellular function. Therefore, dietary recommendations for a variety of high phytonutrient-dense foods might be beneficial as part of individually tailored advice due to the specific roles of these compounds in various organ systems and how they impact intracellular physiology. 

The recognition of the complexity of both an individual and a food may relate to the plethora of conflicting research studies on dietary components and food consumption, whether saturated fat, cholesterol, eggs, coconut oil, or caffeine. It is difficult for nutrition researchers to take into account the complexity of how a food interacts with the multitude of variables in a single individual, including, but not limited to, genotypic or epigenetic stratification, two aspects closely associated with an individual's requirements. Part of the reason for this omission may be due to the lack of adequate diagnostics, difficulty with interpretation, or the paucity of long-term, prospective studies to demonstrate how to clinically integrate this information with therapeutics.

Despite the opportunities that lie ahead for developing a sophisticated interface between technology, metrics, and nutritional interventions, several instances of personalized nutrition approaches have begun to emerge in the literature, suggesting that the introduction of personalized lifestyle medicine is perhaps timely and appropriate at this point in the evolution of medicine.

- *Iron Need and Iron Transport Polymorphisms.* Most individuals with hereditary hemochromatosis could theoretically be evaluated for mutant genotypes that are associated with primary iron overload and increased transferrin saturation and/or serum ferritin levels for better and faster treatment [13].
- *Zinc Need and Polymorphisms.* Select polymorphisms in interleukin-6 and metallothionein may alter one's need for dietary zinc [14].
- *Vitamin D Requirement for Diabetics with Polymorphisms in the Vitamin D Receptor.* Variations in the vitamin D receptor may influence vitamin D requirement and utilization in individuals with type 2 diabetes [15].
- *The Influence of Polymorphisms on Coenzyme Q10 (CoQ10) Need for Energy Production and Its Role in Cerebellar Ataxia.* Genetic variants in the biosynthesis, reduction, and metabolism have been shown to be correlated with plasma CoQ10 levels [16]. CoQ10 deficiency genes were sequenced in patients with unexplained ataxia. CABC1/ADCK3 mutations were identified in symptomatic patients along with decreased muscle concentrations of CoQ10 [17].
- *Folate, MTHFR Polymorphisms, and Depression.* A number of studies have demonstrated the association between low serum folate levels and incidence of depression with a higher frequency of genetic variations in the methylene tetrahydrofolate reductase (MTHFR) enzyme in depressed versus nondepressed individuals [18]. It has been suggested that MTHFR genotyping may be helpful in determining which patients would benefit from L-methylfolate supplementation to override the limitation of reduced conversion of folic acid to this biologically active form due to the polymorphism in MTHFR [18].
- *B Vitamin Gene Variants and Risk to Smoking-Induced Lung Cancer.* Identification of polymorphisms in B vitamin metabolism, particularly folate, has been associated with lung cancer risk, thereby potentially providing an individualized strategy to nutritional interventions for smokers [19].
- *Antioxidants and Polymorphisms in Glutathione S-Transferases (GST).* Propensity towards increased oxidative stress and inflammation can be partially determined by GST genotype. Of all the nutritional factors measured, serum vitamin C was the most consistent nutrient associated with genetic variants of GST [20].
- *Bitter Tasting and Body Composition Differences.* The individual's ability to taste bitter compounds is highly variable and depends on structural and genetic differences in 25 human bitter receptors, termed T2Rs [21]. Since there appears to be a relationship between bitter tasting ability and body mass index (BMI) [22], assessment of a patient's ability to taste bitter may be a useful individualized tool for understanding modifications in metabolism and determining whether inclusion of bitter compounds in the diet or through supplemental means is warranted.

Here are additional examples of clinical conditions for personalized lifestyle medicine interventions:

- salt restriction for subtype of hypertension;
- dietary cholesterol restriction for subtype of hypercholesterolemia;
- dietary saturated fatty acid intake for apoE4;
- gluten restriction for grain intolerance;
- fructose restriction for fructose intolerance;
- lactose restriction for lactose intolerance;
- fava bean restriction for favism;
- carbohydrate restriction for glycogen storage disease;
- folate supplementation of MTHFR polymorphisms;
- lutein supplementation for risk to macular degeneration;
- vitamin A supplementation for poor converters of beta-carotene;
- vitamin D supplementation for individuals who are not capable of efficient or sufficient hydroxylation;
- sulfate supplementation for individuals who not capable of efficient or sufficient sulfation;
- CoQ10 supplementation for statin-induced myopathy;
- dietary biogenic amine restriction for poor metabolizers (phenylethylamine);
- increased long chain omega 3 fatty acids for poor delta 3 dehydrogenase and elongase activities;
- carnitine supplementation for poor acyl-carnitine biosynthesis;
- biotin supplementation for specific bone and heart related functions;
- glycine supplementation for reduced detoxification capability;
- arginine supplementation for reduced eNOS activity;
- N-acetylcysteine supplementation for poor glutathione biosynthesis;
- branched chain amino acid supplementation for individuals with sarcopenia;
- magnesium supplementation for antihypertensive meds;
- iron restriction for hemochromatosis;
- iron supplementation for iron “wasters”;
- selenium supplementation for reduced GSH peroxidase activities;
- DHEA for steroidogenesis deficiencies;
- graded aerobic exercise for those with exercise intolerance;
- ketogenic diets for epilepsy;
- higher protein diets for later stage insulin resistance;
- low protein diets for chronic renal disease.

### 3.1. Dietary Soy (Isoflavones) and Cancer

Presently, one of the most controversial areas in nutrition research is whether dietary soy intake confers a beneficial or detrimental effect in individuals with a propensity towards estrogen-sensitive cancers due to the phytoestrogenic activity of soy isoflavones. Many of the positive epidemiological studies investigating soy (isoflavone) intake and breast cancer have focused on the Asian population, and, therefore, there has been some concern as to whether (a) American women respond similarly to soy and (b) whether the type of soy consumed in different countries is comparable. In a recent review article assessing soy intake in women with breast cancer, Magee and Rowland [23] suggest that the latest research indicates that dietary soy may have differential effects, depending on tumor type. Furthermore, there may be significant interactions between polymorphisms in genes associated with breast cancer, particularly MDM2 and CYP1B1, and dietary soy isoflavone intake. Another aspect of this evolving research is the role of soy isoflavones on epigenetic mechanisms, which is becoming elucidated. From their review of the literature, they conclude: “Recent research suggests that women who are at increased risk of breast cancer due to polymorphisms in genes associated with the disease may especially benefit from high soy isoflavone intake.” Therefore, although the research remains inconclusive, it would seem that more data are required to better assess how a patient's genotype and SNPs could be determined with respect to whether or not they should eat soy products.

### 3.2. Food Intolerances

It has been observed that food intolerances and/or sensitivities are at an all-time high. There seems to be heightened vigilance in the general patient community about the intake of several foods and their corresponding constituents which may cause physiological symptoms within hours after consumption, including sulfites, lactose, cow's milk casein, phenylethylamine (PEA) in chocolate, histamine in fish, and gluten. More than twenty years ago, Hunter [24] proposed that food intolerances were indications that there was an underlying fermentation disorder within the gastrointestinal tract which resulted in the production of metabolites that could not properly be detoxified by liver enzymes in certain individuals with hepatic enzyme polymorphisms. Using a functional assessment to evaluate intestinal microflora, Valeur et al. [25] correlated food hypersensitivity and abdominal symptoms with higher proportions of n-butyric acid. In-depth studies are required to examine the interrelationship between food intolerances, the immune system, enzyme deficiency or inadequacy, and detoxification of colonic bacteria-generated metabolites.

Despite the etiology, the fact is that the existing overarching dietary recommendations are simply not practical and applicable for the growing segment of individuals with reactions to common foods. One instance is the worldwide increased prevalence of gluten spectrum disorders and celiac disease over the last 50 years and an increase in the rate of diagnosis of celiac disease in the last decade [26]. Individuals with these disorders, especially celiac disease, must abstain completely from gluten-containing foods to experience symptom relief, especially from grains where there is a predominance of gluten. However, several dietary recommendations advocate the consumption of relatively copious amounts of grains, especially the previous USDA Food Guide Pyramid (which has been subsequently replaced with MyPlate). Moreover, there are indications that gluten intolerance and celiac disease may be associated with neurological disorders such as multiple sclerosis [27–30], ataxia [26–38], dementia [39–41], seizure disorder [41], cognitive impairment [42], and neuropathy [43].

### 3.3. Omega-3 Fatty Acids and APOE

Another somewhat conflicting area of nutrition is that of omega-3 fatty acids, which are touted for their anti-inflammatory effects; however, their effects on plasma lipids have been inconsistent. The American Heart Association Nutrition Committee statement on omega-3 fatty acids [44] concluded from epidemiological and clinical trial research that intake of these essential dietary fats reduced the incidence of cardiovascular disease (CVD), with EPA + DHA supplementation ranging between 0.5 and 1.8 g/day (as fish or supplements) to reduce cardiac and all-cause mortality, specifically. These findings translate into the final recommendation of including at least two servings of fish per week (particularly fatty fish) in addition to the inclusion of vegetable oil and food sources of alpha-linolenic acid. High daily doses (up to four grams) of omega-3 fatty acids are indicated in hypertriglyceridemia.

In the context of these recommendations, consider the emerging literature on the APOE genotype modification of response to EPA and DHA effects on plasma lipid levels [45]. Liang et al. [45] determined APOE genotype status, plasma EPA and DHA levels, plasma lipids and lipoprotein subclass particles in 2340 participants, and reported significant gene-EPA/DHA interactions with HDL-C and lipoproteins. In a smaller, prospective clinical trial with 38 healthy, normolipidemic men who had been assessed for APOE genotype, Olano-Martin et al. [46] evaluated supplementation of EPA-rich oil (3.3 g EPA/day) and DHA-rich oil (3.7 g DHA/day). A significant interaction between DHA supplementation and E4 carriers was noted through the observed increase in total cholesterol, most likely due to the 10% rise in LDL-C. Consequently, it may be worthwhile for healthcare practitioners to determine APOE status before advocating high-dose supplementation, particularly of DHA, to E4 carriers.

### 3.4. Sodium Restriction and Hypertension

Salt restriction is a common dietary recommendation for individuals with hypertension despite the fact that there are well-known heterogenous responses to dietary salt intake. Varying degrees of salt sensitivity exist with modest reductions of intake in some individuals resulting in an immediate decrease in blood pressure while others are salt resistant. The question has been raised as to whether dietary sodium restriction is universally beneficial [47]. It has been suggested that there is a need to distinguish between individuals who would respond to sodium restriction versus those who do not, but there are no available symptomatic assessments or standardized genotypic analyses to provide the clinician with data they need to tailor the dietary recommendation to reduce sodium to their patients. Specific gene variants associated with salt sensitivity have been identified; however, translation to the clinical setting is lacking. Common genetic variants of the kallikrein-kinin system have been explored with relationship to salt sensitivity [48]. It was determined that genetic variants of the bradykinin receptor B2 gene (BDKRB2) and the endothelin converting enzyme 1 gene (ECE1) were significantly associated with salt sensitivity in 1,906 Han Chinese subjects [48]. In addition to the genetic variants, Rebholz et al. [49] has indicated that salt sensitivity of blood pressure may be influenced by environmental factors such as degree of physical activity, yet another factor to consider in a personalized approach to blood pressure reduction.

### 3.5. Dietary Cholesterol and Hypercholesterolemia

Limitations on dietary cholesterol have been proposed by the American Heart Association of no more than 300 milligrams daily for healthy Americans due to the perception that its intake is associated with increased risk for coronary heart disease (CHD) [50]. On the contrary, other countries, such as Canada, Australia, New Zealand, Korea, and India, have not established upper limits for dietary cholesterol intake. Fernandez [50] and Kratz [51] have questioned whether such a dietary restriction should be imposed as a general guideline considering that current epidemiological evidence does not support the correlation between dietary cholesterol and increased CHD risk. About one-quarter of the population is sensitive to dietary cholesterol and responds with increased plasma LDL; however, this elevation is accompanied by a compensatory rise in HDL-cholesterol, resulting in no significant change in the LDL/HDL cholesterol ratio, a marker of CHD risk. Moreover, as Fernandez [50] states, dietary cholesterol may be instrumental in reducing another CHD risk factor, levels of small, dense LDL particles. Further, a recent meta-analysis of prospective cohort studies by Rong et al. [52] investigating the dose-response relationship between egg consumption and risk of CHD and stroke reported no evidence of an association between higher consumption of eggs (up to one egg daily) and CHD or stroke. However, there was a subgroup of diabetic patients that responded with increased risk due to higher egg consumption.

## 4. Personalized Lifestyle Recommendations: Application to Physical Activity

With respect to lifestyle recommendations, there is increasing evidence and advocacy from science, industry, and government leaders that the national guidelines of increasing overall physical activity, preferably to a minimum of 150 minutes of moderate activity per week, may require some degree of personalization [53]. There are multiple questions that arise from this general activity recommendation, including the following.

- Are all forms of activity equal for every person?
- Could maximum benefit be achieved through moderate versus high-level exertion?
- Are there differences between individual and group responses to activity?
- What is the optimal duration and intensity for various genotypes and phenotypes?
- Should there be multimodal interventions or the implementation of a single activity only?

Some publications have begun to appear on exercise genomics and its potential application [54]. The identification of specific genetic variants in the functionality of skeletal muscle metabolism and strength may be useful for promotion of better exercise tolerance in clinical conditions such as McArdle disease. In a recent study by Williams and Thompson [55], type and intensity of exercise were examined in two large cohorts of runners (*n* = 33 060) and walkers (*n* = 15 945) with relationship to coronary heart disease (CHD) risk factors. They concluded that moderate walking and vigorous running led to similar risk reductions for major chronic diseases. Moreover, a review of recent findings in exercise genomics includes Rankinen et al. [56] who identified that nine specific SNPs largely accounted for the heritability of submaximal heart rate training response. Ahmetov et al. [57] have suggested that the endurance athlete may exhibit a cluster of genetic factors within metabolic pathways related to proportion of slow-twitch muscle fibers and maximal oxygen consumption that may translate into an “elite phenotype”. 

Additionally, it may be worthwhile to examine how combinations of lifestyle medicine factors, such as gene variants, exercise, and disease state, may interact to provide more information to the patient. Hagberg et al. [58] documented the gene-exercise interactions with relation to improved insulin sensitivity, the MTHFR gene correlation with carotid stiffness in unfit individuals, and associations between the C-reactive protein gene with training-induced alterations in left ventricular mass. Hence, an individual's genetics may play a significant role in their response to exercise as has been discussed in the recent publications on exercise genomics [58]. Also, there may be individualized responses not only to physical activity due to one's genotype, but also compounded interactions between serum lipids, exercise, and SNPs.

## 5. Personalized Lifestyle Recommendations: Application to Stress and Behavior

Although stressors have been estimated to be a major contributor to chronic disease development and propagation, there is less emphasis on stress modulation within public health recommendations compared with those of diet and exercise. The ability of a patient to modify their behavior and reaction to stressors is a crucial aspect to consider in the context of personalized lifestyle medicine. It is conceivably difficult to successfully implement lifestyle changes in diet or activity unless there is an underlying adjustment in behavior and locus of control as it relates to stressors. Therefore, taking the essential behavioral aspects of lifestyle medicine into account is much needed to fortify the physical changes that are required for health. An example of a successful comprehensive lifestyle medicine intervention utilizing stress management and group support meetings is reflected in the several decades of clinical research employing this multifaceted regimen into different patient groups by Dean Ornish [59].

Similar to nutrition and exercise, there is a role for personalization in one's approach to stress, which can be encompassed in the diversity of modalities that an individual can choose from to modify their behavior to help them cope with stress, including mind-body medicine practices which are known to balance the parasympathetic and sympathetic nervous systems such as the Relaxation Response developed by Herbert Benson, Mindfulness-Based Stress Reduction (MBSR) from Jon Kabat-Zinn, meditation, yoga, and diaphragmatic breathing. While the basic physiological mechanisms of these practices have been postulated for years, the underlying biological and genetic pathways mediating these effects have not been well researched. Studies in the area of mind-body medicine are gradually becoming more molecular focused.

For instance, Dusek et al. [60] compared gene expression patterns between subjects with long-term relaxation response training and those who were novices. Between the two groups, there was a statistically significant difference in expression of 2,209 genes related to cellular metabolism, oxidative phosphorylation, generation of reactive oxygen species, and response to oxidative stress. Furthermore, Black et al. [61] found that a yogic meditation for 12 minutes daily for 8 weeks prescribed to 39 caregivers resulted in significant changes in genome-wide transcriptional profiles; 68 genes were differentially expressed after adjusting for confounding variables with upregulated genes including immunoglobulin-related transcripts and downregulated transcripts for proinflammatory cytokines. 

Certain individuals may be epigenetically and genetically more inclined to respond to stress. Animal studies have indicated that there are epigenetic alterations in specific brain regions in response to licking and grooming behaviors of pups by rat mothers. Specifically, Zhang et al. [62] reported that decreased frequency of pup licking and grooming resulted in increased methylation of the exon 17 glucocorticoid receptor promoter in the hippocampus, leading to increased hypothalamic-pituitary-adrenal (HPA) responses to stress. Similarly, in humans, children subjected to abuse have decreased hippocampal expression of the glucocorticoid receptor and heightened stress responses. Therefore, parental influences during offspring development are instrumental in forming an imprint for the future adult in regulation of the stress response. The early stressed offspring must learn to adapt to environmental challenges and maintain stability through change, a measurable characteristic referred to by Karatoreos and McEwen [63] as “resilience.” Glucocorticoids can continue to modify the structure and function of neural circuits throughout an individual's lifetime, a process referred to as allostasis, or the process in which the individual regains stability. It has been suggested that through the influence of allostatic modulators, the brain can become more plastic and potentially mitigate the negative influence of a childhood experience [63].

In addition to epigenetic effects and continual allostatic adjustments, a number of genetic variants have been reported to cause altered responses to stress and mood disorders including genes of the serotonin transporter and endocannabinoid CB1 receptors [64]. For instance, polymorphisms in the catechol-O-methyltransferase enzyme have been implicated in human mental illness with the presence of certain SNPs resulting in increased susceptibility to posttraumatic stress disorder (PTSD) [65]. Additionally, Petersen et al. [66] found that there is a predictive interaction between serotonin transporter gene polymorphisms and anxious/depressed symptoms in adolescents, with a stronger relationship noted in late compared with early adolescence. The presence of polymorphisms and the resulting behavior can modify symptoms, such as in dermatological disorders like atopic dermatitis, which can be aggravated by anxiety [67]. One variant of the serotonin transporter gene has been shown to be associated with high-anxiety traits and correspondingly increased atopic dermatitis [67].

## 6. Personalized Lifestyle Recommendations: Application to Environment and Toxin Load

Another important aspect to consider in personalized lifestyle medicine recommendations is to consider the influence of human exposure to environmental toxins on health, especially since 90% of the risks of chronic disease are due to nongenetic factors [68]. It would seem that the environment of the individual would have much influence on one's ability to develop health or disease [68–70]. Thus, the concept of the “exposome” as defined by Rappaport [68] to be the totality of exposures accrued by an individual over their life span could be relevant in compiling a personalized approach to one's health. There are at least two sources of toxicants to quantify: the influx of exogenous sources of toxic chemicals from air, food, water, drugs, and radiation; and internally generated metabolites from processes such as inflammation, lipid peroxidation, oxidative stress, disease states, infections, and microflora.

Increased exposure to external pollutants is strongly associated with the incidence of chronic disease. Several studies have documented the correlation between air pollution and increased cardiovascular morbidity and mortality [71]. Gong et al. [71] used gene-expression profiling to show the interrelationship between diesel exhaust particles and oxidized phospholipids in upregulating pathways implicated in atherosclerosis. Furthermore, compelling evidence is emerging for the role of persistent organic pollutants (POPs), such as polychlorinated biphenyls, dioxins, and pesticides, in chronic diseases such as type 2 diabetes [72–75] by impacting beta-cell function [72], insulin signaling and secretion [73], and mitochondrial function [74], as well as in the development of obesity by influencing adipocyte differentiation and neural circuits that control eating behavior (these toxicants have also thus been termed “obesogens”) [76]. Porta et al. [77] analyzed serum concentrations of 19 POPs in 919 people in Spain and found that more than half of the population studied had concentrations in the top quartile of ≥1 POPs.

Moreover, an individual's ability to metabolize environmental intoxicants, independent of origin, via the liver through the cytochrome P450 family of enzymes, in addition to the accessibility of secondary detoxification pathways including conjugation with glucuronic acid, sulfate, glutathione, amino acids such as taurine and glycine, acetic acid, and methyl groups, must be evaluated through indirect or direct genotypic means for a better understanding of the robustness of their excretion of these toxicants. Drug response variability due to SNPs in the hepatic cytochrome P450 oxidase family of enzymes has been well defined for several decades, although new SNPs continue to be identified [78]. Furthermore, the conjugation pathways mentioned earlier may have genetic variants resulting in altered activity. Sirivarasai et al. [79] demonstrated that increased serum lead levels confers higher C-reactive protein and systolic blood pressure levels; however, there are varying degrees of these biological responses based on the different polymorphisms in GST.

## 7. Transition into a Global System of Personalized Lifestyle Medicine

**Figure 3:**
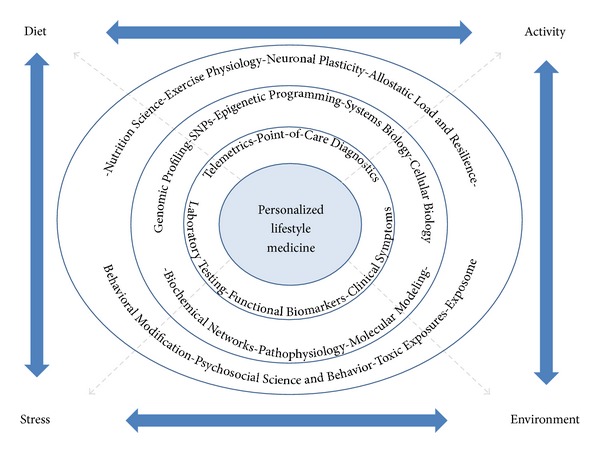
What is personalized lifestyle medicine?

Several systems and medical delineations have been conceptualized in the past decades (Table 1). Personalized lifestyle medicine presents a system of medicine which merges technological advances with the traditional foundation of lifestyle through the psychosocial-behavioral interface (Figure 3). It would seem that with the advent of personalized medicine and the emergence of diagnostics to assess one's genotype and moment-by-moment biomarkers that dietary and lifestyle recommendations will inevitably become individualized to the patient. There have been differing views expressed on whether or not offering personalized nutritional advice based on an individual's genes and SNPs is welcomed by the public at large [80]. As Görman et al. [80] suggest, the evidence for sufficient guidance based on genes and even epigenetics is rather limited and gaps in knowledge need to be overcome; however, the merit in this approach, when scientific data are available, is that it may result in improved compliance and support the individuality and inherent choices to be made by the patient. Nielsen and El-Sohemy [81] used surveys to assess awareness of genotype testing and genotype-based personalized dietary advice or general dietary advice in 149 individuals between the ages of 20 and 35 years. They concluded that a majority of these individuals found dietary recommendations based on genetics to be more useful than general dietary advice.

The delivery of personalized advice requires accurate measurements on one's physiology through genomic analysis and molecular diagnostics. Tailored biomarkers, molecular imaging, rapid assessments through point-of-care devices, telemedicine, and individualized therapeutic treatments are gradually replacing the standardized, “one size fits all” form of medicine, ultimately providing the best form of care, reducing costs, and enhancing safety by limiting side effects. To date, there have been a number of functional biomarkers introduced into the clinical setting which illustrate the progression of personalized medicine, including the following.

1. The addition of high-sensitivity C-reactive protein as part of a laboratory panel to assess inflammation, particularly as part of the diagnosis of cardiovascular disease and even periodontal disease, is the first. Several polymorphisms have been identified that correlate with increased levels of the inflammatory cytokine, interleukin-6 [82].
2. The utilization of the liver enzyme marker, gamma-glutamyl transferase (GGT), as not just a marker of alcoholism, but an indicator of xenobiotic exposure and increased usage of hepatic glutathione, even at high normal levels, which can be of concern for individuals with polymorphisms in GST, is the second.
3. Investigation of homocysteine levels as a potential risk factor for CVD in addition to its elevation as a possible indicator of methylation inefficiency due to genetic variants in MTHFR is the last.

As increasing recognition for the use of biomarkers as indirect or direct indicators of not just symptoms, but the underlying causes related to an individual's genotypic profile, there will be greater emphasis on a personalized approach to health. Additionally, with the advent of technologies to assist in these types of laboratory measures in becoming mainstream, lifestyle medicine areas, including diet, physical activity, stress responses, and environmental factors, will begin to merge with the outcomes of these tests, resulting in clinicallyapplied personalized lifestyle medicine approaches to most favorably address a patient's condition. In so doing, the patient will be able to regain control of their health and feel empowered in their decisions concerning their outcomes.

For several years before disease onset, a patient may have subclinical manifestations of a disease, indicated by low and/or high normal laboratory values, and the presence of ill-defined symptoms which do not classically qualify for a determined diagnosis. Personalized lifestyle medicine can be integral throughout a patient's life, from prevention to preclinical symptoms to disease manifestation and progression.

Personalized lifestyle medicine encompasses a broad array of disciplines in order to effectively prevent and treat disease, including the interface of technological advances with modern medicine discoveries for eventual dissemination into clinical medicine approaches.

## Figures and Tables

**Table 1:** Differentiation among medical systems.

Preventative medicine	Lifestyle medicine	Personalized medicine	Functional medicine	Personalized lifestyle medicine
(i) Public health recommendations from opinion leader organizations (ii) Generalized guidelines (iii) Focused on prevention rather than treatment	(i) Could be preventive or therapeutic guidelines (ii) Encompasses a broad set of therapies related to one's living and behavioral patterns (iii) Practiced by wide range of medical professionals	(i) Focused on therapeutic strategies involving individualized pharmaceutical prescriptions for improved safety and efficacy (ii) Genomics as foundational tool (iii) Investigational and research driven (iv) Practiced by physicians (v) Patient-centered treatment	(i) Operational system developed to assess a patient's etiology and to devise a treatment protocol to address underlying causes (ii) Composed of multiple heuristics to lead the process of the therapeutic encounter (iii) Involves training and certification (iv) Focus on disease origin	(i) Next generation of medicine (ii) Tailored lifestyle medicine prescriptions directed to the individual based on diagnostics, including genomics and epigenetic information (iii) Uses functional medicine as the underlying operating system but requires the integration of other medical systems (iv) Patient-centered outcomes
